# Functional Reorganization of the Locomotor Network in Parkinson Patients with Freezing of Gait

**DOI:** 10.1371/journal.pone.0100291

**Published:** 2014-06-17

**Authors:** Brett W. Fling, Rajal G. Cohen, Martina Mancini, Samuel D. Carpenter, Damien A. Fair, John G. Nutt, Fay B. Horak

**Affiliations:** 1 Department of Neurology, School of Medicine, Oregon Health & Science University, Portland, Oregon, United States of America; 2 Department of Behavioral Neuroscience, School of Medicine, Oregon Health & Science University, Portland, Oregon, United States of America; 3 Department of Psychiatry, School of Medicine, Oregon Health & Science University, Portland, Oregon, United States of America; 4 Department of Psychology and Communication Studies, University of Idaho Moscow, Idaho, United States of America; 5 Portland VA Medical Center, Portland, Oregon, United States of America; Tianjin Medical University General Hospital, China

## Abstract

Freezing of gait (FoG) is a transient inability to initiate or maintain stepping that often accompanies advanced Parkinson’s disease (PD) and significantly impairs mobility. The current study uses a multimodal neuroimaging approach to assess differences in the functional and structural locomotor neural network in PD patients with and without FoG and relates these findings to measures of FoG severity. Twenty-six PD patients and fifteen age-matched controls underwent resting-state functional magnetic resonance imaging and diffusion tensor imaging along with self-reported and clinical assessments of FoG. After stringent movement correction, fifteen PD patients and fourteen control participants were available for analysis. We assessed functional connectivity strength between the supplementary motor area (SMA) and the following locomotor hubs: 1) subthalamic nucleus (STN), 2) mesencephalic and 3) cerebellar locomotor region (MLR and CLR, respectively) within each hemisphere. Additionally, we quantified structural connectivity strength between locomotor hubs and assessed relationships with metrics of FoG. FoG+ patients showed greater functional connectivity between the SMA and bilateral MLR and between the SMA and left CLR compared to both FoG− and controls. Importantly, greater functional connectivity between the SMA and MLR was positively correlated with i) clinical, ii) self-reported and iii) objective ratings of freezing severity in FoG+, potentially reflecting a maladaptive neural compensation. The current findings demonstrate a re-organization of functional communication within the locomotor network in FoG+ patients whereby the higher-order motor cortex (SMA) responsible for gait initiation communicates with the MLR and CLR to a greater extent than in FoG− patients and controls. The observed pattern of altered connectivity in FoG+ may indicate a failed attempt by the CNS to compensate for the loss of connectivity between the STN and SMA and may reflect a loss of lower-order, automatic control of gait by the basal ganglia.

## Introduction

Balance and gait are impaired in the majority of older people, especially those with age-related, neurological degeneration such as Parkinsonism. Advanced PD is often accompanied by freezing of gait (FoG), a transient inability to initiate or maintain stepping [1]. Historically, freezing episodes have typically been reported in relation to repetitive lower limb movements; however, an emerging body of literature also reports *upper limb* freezing within these same patients [2]. Freezing of the upper and/or lower limb(s) significantly impairs mobility and limits independent function, but the neural underpinnings of freezing episodes remain poorly understood. A growing body of work suggests that these deficits in upper and lower limb movements require increased cognitive control of previously automated actions [3]. Recent work suggests that dysfunction of top-down inhibitory control may serve a critical role in disorders of movement initiation observed in AHDH, Huntington disease, schizophrenia and PD [4].

Anticipatory postural adjustments are crucial for gait initiation prior to voluntary movement and are mediated by the supplementary motor area (SMA) [5], [6], [7]. Impairment in inhibiting or blocking a stepping response until postural preparation is complete may be responsible for ‘start hesitation’ FoG in PD. Progressive dysfunction of circuits involving the SMA has previously been implicated in impaired step initiation in FoG associated with later stages of PD [6], [8]. In particular, inhibition of the SMA with repetitive transcranial magnetic stimulation altered the timing of anticipatory postural adjustments, and this effect was larger the more severe the PD [6]. These data indicate FoG may be related to dysfunction of linking step initiation with postural weight shifts.

In addition to the SMA, multiple subcortical regions are involved in locomotion, specifically the subthalamic nucleus (STN) and the mesencephalic and cerebellar locomotor regions – (CLR and MLR, respectively) [9]. Interestingly, these are all principal areas that demonstrate metabolic increases with disease progression implicating these key motor and cognitive hubs as markers of disease progression in PD [10]. Our recent work demonstrated reduced structural connectivity between the pedunculopontine nucleus (a principal nucleus of the MLR) and the CLR, STN, and SMA, along with multiple regions of the frontal and prefrontal cortex in the right hemispheres of Parkinson’s patients with FoG [11]. Furthermore, Shine and colleagues [12] reported *decreased* neural activity within the STN and the MLR in PD patients during freezing episodes, the magnitude of which was positively correlated with the severity of clinical FoG. Conversely, Snijders et al. [13] reported *increased* activity within the MLR of PD patients who experience FoG during motor imagery of normal walking, i.e. in-between freezing episodes.

Whereas these task-based functional MRI studies provide isolated regions of neural activity, resting state functional connectivity analysis allows for integrative assessments of distributed neural systems. A limited body of work demonstrates functional connectivity differences within PD patients who experience freezing episodes compared to those who do not [14]; however, functional connectivity within the locomotor network has yet to be explored. Taken together, these results implicate compromised structural integrity and potential functional disconnection between subcortical and cortical regions of the locomotor network in individuals who experience FoG; however, functional connectivity within the locomotor network has yet to be explored.

In the current study, we use a novel multimodal neuroimaging approach to assess differences in functional and structural connectivity of the locomotor network between PD patients with (FoG+), those without (FoG−) freezing of gait, and age-matched healthy controls. We hypothesized that FoG+ patients would have reduced functional and structural connectivity within the locomotor network compared to both FoG− and control participants. Furthermore, we predicted that these declines in structural and functional connections would be strongly related to freezing severity.

## Materials and Methods

### Standard Protocol Approvals, Registration, and Patient Consents

Oregon Heath & Science University Institutional Review Board approved this study and all participants gave their informed, written consent before beginning the experiment.

### Subjects

Data were collected from 26 patients with PD and 15 age-matched controls. Participants were recruited through the Parkinson’s Center of Oregon clinic at Oregon Health & Science University (OHSU). Individuals were excluded if they could not safely walk 20 feet without walking aids, or if they had a joint replacement, musculoskeletal or vestibular disorder, dementia, claustrophobia, severe tremor, or metal in their bodies. Due to violation of strict movement thresholds during functional imaging (described below) eleven patients with PD and one healthy control were excluded from further analysis; thus, data presented are from fifteen patients with PD (14 male; age 64.7±6.0 years; duration of disease 9.1±6.2 years) and fourteen healthy controls (5 male; age 66.9±7.8 years). Of the fifteen PD patients, eight were classified as FoG+ based on a score of >3 on the new freezing of gait questionnaire (NFOGQ) [15]. Seven patients scoring ≤3 were classified as FoG−. All PD patients were tested in the “OFF” state, after 12–18 hour overnight withdrawal from anti-parkinsonian medications to mitigate the pharmacological effects on neural activity. Diffusion imaging data of the entire study population has previously been reported [11].

### Freezing of Gait Assessments

All participants provided self-rated assessments of their freezing severity by filling out the NFOGQ [15]. Recent work has called into question the accuracy of such self-rated measurements [16] so we also used more objective clinical assessments of freezing. Because FoG is notably difficult to provoke in the laboratory or clinic setting, clinicians examined trials in which participants performed tight clockwise and counter-clockwise turns, a task known to elicit freezing [17]. Participants wore 3 inertial sensors (Opal by APDM, Portland, OR) mounted on the posterior trunk and on each shank while turning in place for 2 minutes under two different conditions: i) *single task*, turning 360° to their right, then turning 360° to their left, and repeating this at their own comfortable pace for 2 minutes, ii) *dual task* (wearing headphones producing a tone in the left or right ear), turning 360° to their left or right depending on the side in which a tone was produced. The power spectral density (PSD) was calculated for each trial from the antero-posterior shank accelerations using a 4 s Hanning window with 50% overlap (using the Welch method). The total power was then normalized to the area under the PSD for each subject. A Freezing Ratio was calculated as the square of the total power in the 3–8 Hz band, divided by the square of the total power in the .5–3 Hz band [18], [19]. Turning trials were also video recorded and two experienced neurologists who specialize in movement disorders subsequently reviewed and provided a clinical measure of freezing severity. Scores were based upon on ordinal scale from 0–4 where: 0 =  absent, 1 =  mild, 2 =  moderate, 3 =  significant interference with movement, and 4 =  severe with risk for falls. The raters were blinded to whether the patients with Parkinson’s disease were identified as having freezing of gait or not based upon their self-assessed NFOGQ score; each patient’s average score and the objective Freezing Ratio in the two turning conditions were used to compare with functional connectivity.

### Image Acquisition

Participants were scanned on a 3.0 T Siemens Magentom Tim Trio scanner with a 12-channel head coil at Oregon Health and Science University’s Advanced Imaging Research Center. One high-resolution T1-weighted MPRAGE sequence (orientation = Sagittal, echo time = 3.58 msec, repetition time = 2300 msec, 256×256 matrix, resolution 1.0×1.0×1.1 mm. total scan time = 9 min 14 sec) was acquired. One BOLD-weighted functional image was acquired with a T2*-weighted EPI (repetition time = 2000 msec, echo time = 30 msec, flip angle = 90°, field of view = 240 mm, 33 slices covering the whole brain, resolution = 3.8 mm^3^, total scan time = 10 min 6 sec). Steady-state magnetization was assumed after three volumes (∼ 6 sec). Subjects were instructed to remain still and fixate on a standard fixation cross, projected in the center of their visual field. Three sets of diffusion-weighted images were collected using a 30-gradient direction, whole-brain echoplanar imaging sequence (TR = 9,100 ms, TE = 88 ms, field of view = 240 mm^2^, b value = 1,000 s/mm^2^, isotropic voxel dimensions = 2 mm^3^) and three images in which the b value was equal to zero. A static magnetic field map was also acquired using the same parameters as the diffusion weighted sequence.

### Functional Image Preprocessing

All functional images were preprocessed in an identical method to best alleviate known types of artifacts [20]. These steps included 1) removal of central spike caused my MR signal offset, 2) correction of odd versus even slice intensity differences due to interleaved acquisition without gaps, 3) correction for head movement within the run, and 4) within-run intensity normalization to a whole brain mode value of 1000. Atlas transformation of the functional data was computed for each individual via the MP-RAGE scan [21]. The functional data was then resampled into atlas space on an isotropic 3-mm grid, combining movement correction and atlas transformation in one interpolation [22]. All subsequent operations were performed on the atlas-transformed volumetric time series.

Connectivity preprocessing followed prior methods [23]. These steps included: 1a) a temporal band-pass filter (0.009 Hz<f <0.08 Hz), 1b) spatial smoothing (6 mm full width at half maximum). All data were computed with and without smoothing; unless otherwise stated, all results reported were drawn from the no smoothing condition, 2) regression of six parameters obtained by rigid body head motion correction, 3) regression of the whole brain signal averaged over the whole brain, 4) regression of ventricular signal averaged from ventricular region of interest (ROI), and 5) regression of white matter signal averaged from white matter ROI. Regressions of the first order derivatives from steps 3–5 were also included in the correlation preprocessing. These preprocessing steps are thought to best reduce variance that is unlikely to reflect neuronal activity [24].

### Motion Correction

In an effort to account for inter-acquisition subject motion that could potentially be problematic for correlation analysis, an additional motion correction step was implemented as described by Power and colleagues [25]. This method, titled framewise displacement (FD), calculates a time series of volume-to-volume motion from the movement measures created by the aforementioned rigid body motion correction. This is calculated for all six parameters by the equation FD*_i_* = |Δ*d_ix_*|+|Δ*d_iy_*|+|Δ*d_iz_*|+|Δα_i_|+|Δβ_i_|+|Δγ_i_|, where Δd*_ix_* = d*_(i_*_−*1)x*_ – d*_ix_*, (the same holds true for the other five parameters [*d_ix_ d_iy_ d_iz_* α*_i_* β*_i_* γ*_i_*]). Essentially, this formula sums the absolute values of the volume-to-volume changes in six directions. The rotational measures were first converted to millimeters by calculating surface displacement on a sphere of radius 50 mm, which is the approximate distance from the cerebral cortex to the center of the head. This method ‘scrubs’ the data by removing any volume that exceeds a set threshold (in this case 0.5 mm) from the time series. The correlation analysis was performed on the remaining concatenated volumes. If greater than 50% of the functional volumes (>150 out of 300) exceeded the threshold, participants were excluded from analysis. Due to excessive movement, one healthy control participant, four FoG− participants, and seven FoG+ participants were excluded.

### fcMRI Region of Interest Selection

**Figure 1:**
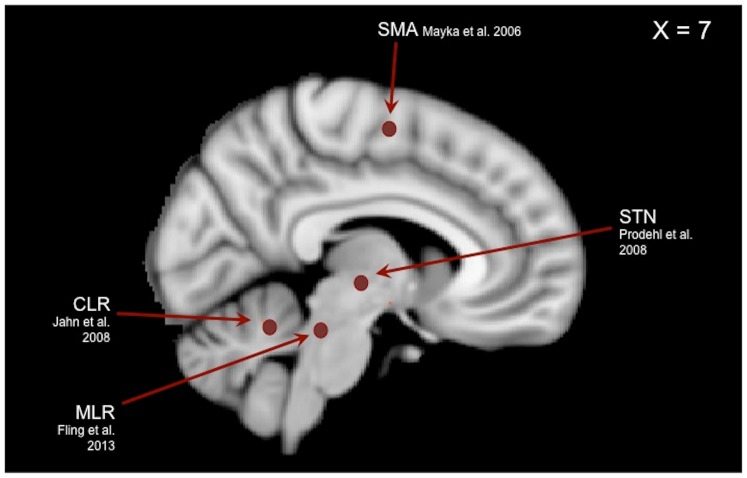
Locomotor hubs used as regions of interest for functional connectivity analysis. X = 7 is chosen for display as it is the only slice on which all ROIs have some overlap. Please refer to the Methods section for coordinates describing the centroid of each sphere.

We selected the SMA, STN, MLR and CLR as locomotor hubs, all of which have previously been identified as neural regions (by chemical or electrical stimulation within the cat) [26], [27], [28] known to initiate and pace gait (**Figure 1**). All regions of interests (ROIs) were analyzed in Talairach space. For those ROIs that were initially identified within MNI space (MLR and CLR), the Lancaster transform was used to convert into Talairach space [29]. A 10-mm sphere was created at the peak coordinates of the SMA on the midline (*x* = 0, *y* = −7, *z* = 55) as identified by activation likelihood estimates identified in the three-dimensional locations and boundaries meta-analysis of motor regions by Mayka and colleagues [30]. For all sub-cortical ROIs a 6-mm sphere was created bilaterally for the STN, MLR and CLR. The STN lies ventral to the thalamus, and lateral and caudal to the hypothalamus. Peak coordinates for the STN (*x* = ±11, *y* = −14, *z* = −3) were chosen from the task-related activation ROI template of the human basal ganglia identified by Prodoehl et al. [31]. The MLR lies ventral to the inferior colliculus, in the upper pons; it comprises the pedunculopontine and cuneiform nuclei. Peak coordinates for the MLR (*x* = ±6, *y* = −30, *z* = −19) were selected similar to our recent work [11], and were informed by local field potential recordings [32] and immunohistochemistry [33]. The CLR is located in the cerebellar midline, slightly dorsal to the fastigial nuclei; peak coordinates (*x* = ±7, *y* = −52, *z* = −16) were identified based on recent imaging of the supraspinal locomotor centers in the brainstem and cerebellum [34].

As discussed in the Introduction, the SMA is thought to contribute to the generation of anticipatory postural adjustments, which act to stabilize supporting body segments prior to movement. Because dysfunction of the SMA has been implicated repeatedly in contributing to altered step initiation, functional connectivity strength was restricted to analyses between the SMA and the bilateral sub-cortical locomotor hubs assessed by correlation coefficients between each ROI pair (e.g., SMA – left STN). The mean time series from the SMA was correlated with the mean time series from each of the six sub-cortical ROIs using Pearson’s coefficient of correlation (r) for each participant. We examined normality of distribution using the Kolmogorov-Smirnov test for all functional connectivity pairs. Assumptions for parametric statistics were met, so we compared the strength of functional connectivity between the SMA and the six sub-cortical ROIs for the three groups (controls, FoG−, FoG+) using a repeated-measures ANCOVA. UPDRS score was included as a covariate for all comparisons. Significant main effects were subjected to post-hoc analyses using a two-tailed Tukey’s HSD test to correct for multiple comparisons. All statistical analyses were carried out using SPSS (IBM SPSS Statistics v. 19). A p-value ≤0.05 was considered significant.

### Diffusion Tensor Imaging Analysis

We used the same methodology as employed in a previous study [11]. Diffusion data were processed using the tools implemented in FSL (Version 5.0; www.fmrib.ox.ac.uk/fslwww.fmrib.ox.ac.uk/fsl). The three raw data sets were first corrected for eddy current distortions and motion artifacts using the correction tool (FDT 1.0), then averaged to improve signal-to-noise ratio [35] and subsequently skull-stripped (using FSL’s brain extraction tool). The principal diffusion direction was estimated for each voxel as a probability density function, using Bayes’ rules in order to account for noise and uncertainty in the measured data. As described elsewhere [36], the implicit modeling of noise in a probabilistic model enables a fiber tracking procedure without externally added constraints such as fractional anisotropy threshold or fiber angle. Thus, fiber-tracking in or near cortical areas becomes more sensitive. The use of a 2-fiber model [37] also improves the modeling of crossing fibers. By sending out 25,000 streamline samples per seed voxel, we mapped the probabilistic connectivity distributions for each voxel in each ROI (see below). For each individual, the fractional anisotropy images were normalized into Montreal Neurological Institute (MNI) space by using a linear (affine) registration and Fourier interpolation through the FMRIB linear image registration tool.

**Figure 2:**
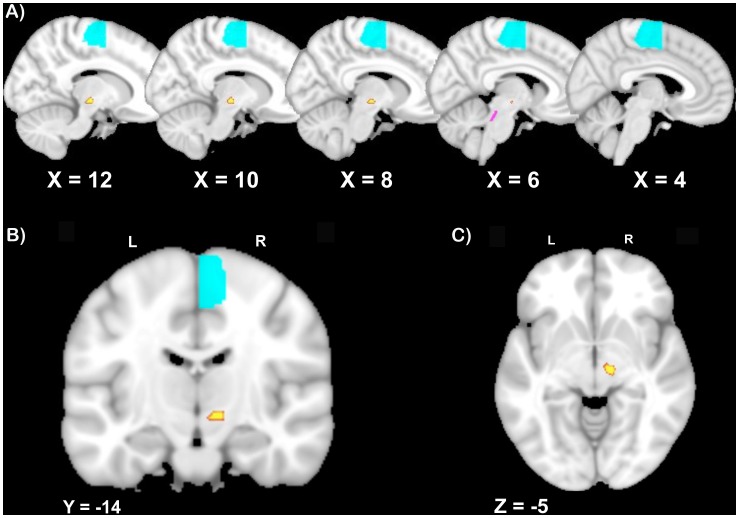
Structural connectivity ROIs in the A) sagittal, B) coronal, and C) axial plane. The SMA, as defined by Mayka et(2006) is shown in blue. The STN, as defined by Prodoehl et al (2008) is shown in yellow. The MLR, as defined by Fling et al. (2013), is shown in purple. All ROIs are overlaid on the MNI 1 mm template and are displayed in neurologic convention (right = right). Similar ROIs for the SMA and MLR were used in the left hemisphere (not displayed here).

We performed probabilistic fiber tractography to assess *structural* connectivity strength within the locomotor network. Due to the lack of a well-defined template of the CLR’s boundaries and the limited data identifying anatomical connections between the CLR and the SMA, we did not attempt to identify fiber tracts connecting these two regions. Thus, probabilistic fiber tracking was initiated from every voxel within the binarized region of interest in each participant’s native diffusion space to delineate the following tracts: (1) rSTN – SMA; (2) lSTN – SMA; (3) rMLR – SMA; and (4) lMLR – SMA. The STN was defined using the human basal ganglia human area template created by Prodoehl et al. [31] and the SMA was defined using the human motor area template as defined by Mayka et al. [30]. The MLR was defined using the template recently described by Fling et al. [11] (See **Figure 2** for ROIs). Seed-masks were determined in MNI space and transformed to subject diffusion space. Streamline samples (25,000) were sent out from each voxel, with a step length of 0.5 mm and a curvature threshold of 0.2. For each connection pair, the number of samples reaching the target mask was divided by the number of voxels in the seed mask, and resulting values were used as an indicator of relative structural connectivity strength across participants [38], [39]. For the structural connectivity pairs we again examined normality of distribution using the Kolmogorov-Smirnov test. Assumptions for parametric statistics were met, so we compared the strength of structural connectivity for the three groups (controls, FoG−, FoG+) using a repeated-measures ANCOVA. For all comparisons, UPDRS score was again included as a covariate. Significant main effects were subjected to post-hoc analyses using a two-tailed Tukey’s HSD test to correct for multiple comparisons. All statistical analyses were carried out using SPSS (IBM SPSS Statistics v. 19). A p-value ≤0.05 was considered significant. Where significant main effects were identified, functional and structural connectivity strength were entered as independent variables for linear regression analysis to identify associations with metrics of freezing including: (1) NFOGQ, (2) Clinical FoG rating, (3) Objective Freezing Ratio and were corrected for multiple comparisons.

## Results

Participant characteristics and freezing assessments can be viewed in **Table 1**. Briefly, no difference was observed in disease severity between FoG+ and FoG−, although there was a trend towards greater severity in the FoG+ group as assessed by UPDRS motor scores (*P*<0.16) and Hoehn and Yahr (*P*<0.15*)*. Patients classified as FoG+ had significantly longer disease duration than FoG−. The objective Freezing Ratio was significantly higher in FoG+ participants compared to FoG−, in both the single- and dual-task conditions (P<0.001). Additionally, neurologists who were blinded to participants’ self-reported FoG status rated freezing as significantly more severe in FoG+ participants than in FoG− participants (*P*<0.001). Inter-rater reliability for neurologist scoring was high (ICC = 0.98), and only one FoG− participant received a rating greater than 0 from the neurologists (a score of 1 from one of the two neurologists).

**Table 1:** Patient characteristics.

	Age	DiseaseDuration	More AffectedSide (R/L/E)	L-Dopa(mg/day)	UPDRSMotor	H & Y	NFOGQ	Clinical FoGRating	Objective FreezingRatio *ST DT*		FrameExclusion (%)
HC (n = 14)	66.9 (7.8)	–	–	–	–	–	–	–	0.3 (0.2)	0.3 (0.2)	19.1 (16.3)
FoG− (n = 7)	63.9 (6.4)	**4.4** (2.5)	3/3/1	504.3 (315.9)	29.6 (9.2)	1.9 (0.9)	**0.4** (1.1)	**0.09** (0.2)	**1.4** (0.9)	**1.5** (1.1)	17.7 (19.5)
FoG+ (n = 8)	65.4 (6.0)	**13.2** (5.5)	3/4/1	625.0 (365.5)	36.3 (7.8)	2.6 (0.7)	**18.1** (5.4)	**2.0** (1.7)	**4.8** (3.1)	**4.6** (3.0)	9.8 (12.1)

Significant differences (*P*<0.05) between PD groups, assessed by Tukey’s HSD test, are highlighted in bold. All data displayed are mean (± standard deviation). HC = healthy controls. R = right, L = left, E = equal. ST = single task, DT =  dual task condition.

### Locomotor Network Functional Connectivity Strength

**Figure 3:**
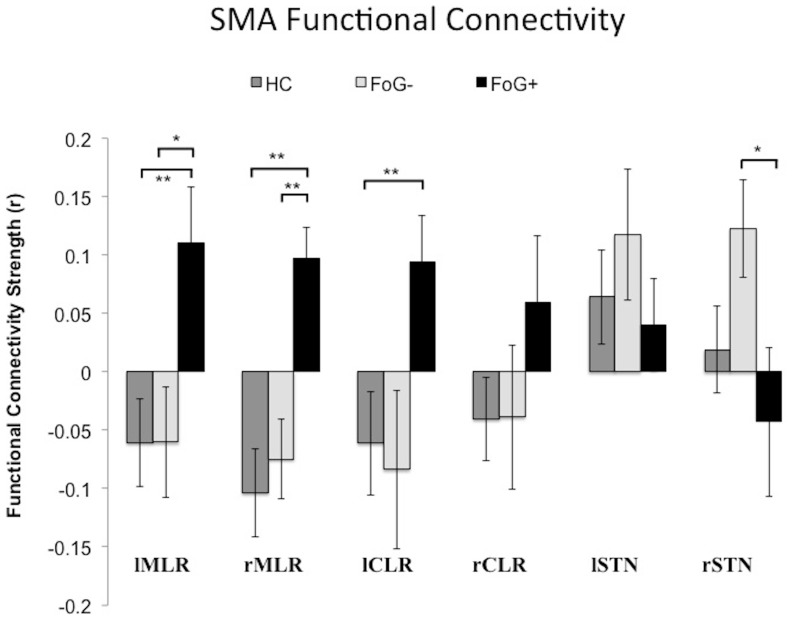
Functional connectivity strength between the SMA and the other locomotor hubs. FoG+ patients show greater connectivity to the MLR and CLR, but reduced connectivity to the STN. **P<0.001, *P<0.05.

No group difference was observed with regards to the percentage of frames excluded for functional runs, and was therefore not included as a covariate in the statistical model (**Table 1**). Group differences were observed for four of the six functional connectivity comparisons: rSTN – SMA (F_2,29_ = 4.2; *P = *0.024), lMLR – SMA (F_2,29_ = 4.9; *P = *0.016), rMLR – SMA (F_2,29_ = 5.2; *P = *0.013), and lCLR – SMA (F_2,29_ = 4.3; *P = *0.026) (see **Figure 3**). No group differences were observed for rCLR – SMA (F = 1.79; *P* = 0.19) or lSTN – SMA (F = 0.776; *P = *0.57). Post-hoc *t-*tests for rSTN – SMA connectivity show significantly stronger communication for FoG− compared to FoG+ (*t = *3.37; *P = *0.006), but not compared to controls (*t = *1.71; *P* = 0.2). No difference was observed between controls and FoG+. Conversely, FoG+ show significantly stronger connectivity between lMLR – SMA compared to both controls (*t* = 2.95; *P* = 0.008) and FoG− (*t = *2.98; *P = *0.012). Similar group differences were found between rMLR – SMA with FoG+ demonstrating greater connectivity than controls (*t = *2.97; *P* = 0.008) and FoG− (*t = *3.2; *P* = 0.008). Finally, connectivity strength between lCLR – SMA was significantly stronger in FoG+ than controls (*t = *3.08; *P* = 0.006), but not significantly greater for FoG+ than FoG− (*t = *1.97; *P = *0.075). For the latter three post-hoc comparisons, no differences were observed between controls and FoG− participants (*P*>0.7).

The overall pattern of these results demonstrated a re-organization of functional communication within the locomotor network in FoG+ patients whereby the SMA communicated with the MLR and CLR to a greater extent than in control participants or FoG− PD patients. We direct the reader to Figure S1 for full cohort analysis without the additional step of framewise displacement motion correction.

### Locomotor Network Structural Connectivity Strength

**Figure 4:**
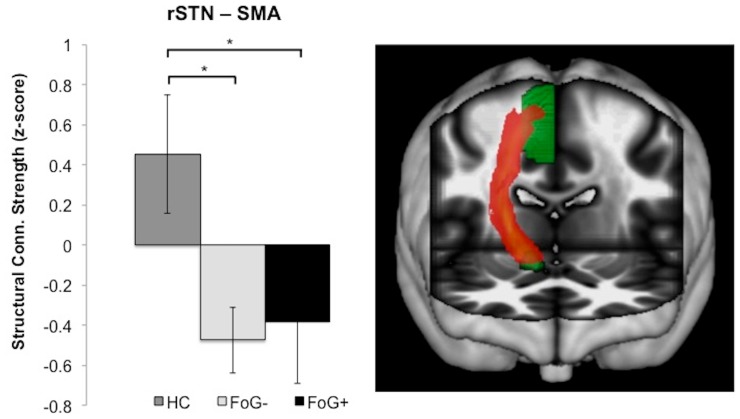
Structural connectivity between the SMA and right STN, as assessed by diffusion tensor imaging. **A)** Normalized z-scores for structural connectivity strength between the rSTN – SMA. HC have significantly greater connectivity than either FoG− or FoG+. **B)** Rendered fiber tracts (red) from one healthy control demonstrating the structural connectivity within the hyperdirect loop between the SMA and STN (both displayed in green). *P<0.05. HC = healthy controls.

A main effect of group was found for rSTN – SMA structural connectivity (F = 4.2; *P = *0.026), but no difference was observed for rMLR – SMA structural connectivity strength (F = 2.4; P = 0.10). Nor were group differences in structural connectivity strength observed within either the lSTN – SMA (F = 0.3; P = 0.72) or lMLR – SMA loop (F = 0.94; P = 0.41). Post-hoc comparisons for the rSTN – SMA loop demonstrate greater connectivity for control participants in comparison to both FoG− (*t* = 2.12; *P* = 0.02) and FoG+ (*t* = 1.83; *P* = 0.04) groups. No difference was observed between the two PD sub-groups (P = 0.8; **Figure 4**). The overall pattern of these results demonstrate similar reductions in *structural* connectivity strength between the rSTN – SMA in both PD sub-groups, despite the notable difference observed in *functional* connectivity of this loop for FoG− compared to FoG+.

### Relationships between Locomotor Network Connectivity and Freezing

**Figure 5:**
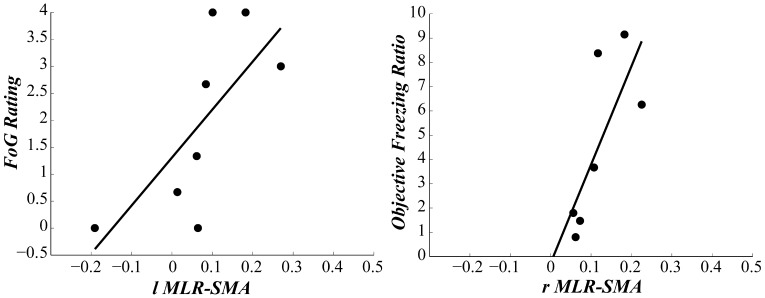
Associations between locomotor network functional connectivity and freezing severity. **A)** Functional connectivity strength of the lMLR – SMA loop was positively correlated with clinical rating of FoG severity during the single task turning condition (r = 0.71). **B)** Functional connectivity strength of the rMLR – SMA loop was also positively correlated with objective sensor measurement of FoG severity during dual-task turning (0.76).

Seed pairings demonstrating group differences in functional connectivity strength were analyzed for relationships with metrics of freezing (see **Table 2** for all relationships). Briefly, higher functional connectivity between the lMLR-SMA pair (Figure 5), as well as between the lCLR – SMA pair, was strongly correlated with higher clinical ratings of FoG and self-reported NFOGQ scores in FoG+. Similarly, the higher functional connectivity between the rMLR – SMA pair was strongly related with higher Freezing Ratio during turning under the dual task condition in FoG+ (Figure 5). It is worth noting that disease duration was not related to functional connectivity strength for any of the identified seed pairings, nor were any significant relationships between structural connectivity strength and measures of freezing found.

**Table 2:** Relationships between functional connectivity strength and 1) self-reported freezing severity, 2) clinical ratings of freezing severity, 3) Objective Freezing Ratio during single task turning, 4) Objective Freezing ratio during dual task turning, and 5) disease duration in FoG+ patients.

		lMLR – SMA	rMLR − SMA	lCLR − SMA	rSTN – SMA
FoG+	NFOGQ	0.74*	0.45	0.63*	0.19
FoG+	FoG Rating	0.71*	0.29	0.37	0.28
FoG+	Freezing Ratio, single task	0.45	0.20	0.04	0.46
FoG+	Freezing Ratio, dual task	0.22	0.76*	0.44	0.86*
FoG+	Dis. Duration	−0.14	0.04	0.19	−0.13

Stronger functional connectivity between lMLR – SMA and lCLR – SMA was related to more severe self-perceived or clinically assessed freezing, while stronger functional connectivity between rMLR – SMA was related to more severe freezing assessed with the Objective Freezing Ratio during the dual task turning. Values represent correlation coefficients (r). Dis. Duration = disease duration. *P<0.05 shown in bold, analyses are corrected for multiple comparisons.

## Discussion

Bipedal locomotion is a complex process involving coordinated supraspinal control between multiple locomotor hubs. These locomotor regions and their contributions to gait have recently been reviewed [9], [40], and a growing body of literature has begun to explore the clinical implications of dysfunction within these neural hubs. The current findings demonstrate a reorganization of functional communication within the locomotor network in FoG+ patients whereby the higher order motor cortex (SMA) responsible for gait initiation communicates with the MLR and CLR to a greater extent than for FoG− or controls. Conversely, FoG− had significantly greater functional connectivity than FoG+ in the hyperdirect pathway between rSTN and SMA. The observed pattern of altered connectivity in FoG+ does not appear to serve a useful compensatory role, but rather may contribute to FoG, as evidenced by the positive association between ratings of FoG severity and increased functional connectivity in the MLR – SMA and CLR – SMA loops. Our results complement a growing body of literature indicating that age- and disease-related deficits in upper and lower limb movements are reflective of an increased need for cognitive, or higher-order motor control of previously automated actions [41].

### Locomotor Network Functional Connectivity

Neural regions underlying locomotor function have traditionally shown diminished functional activation in patients with PD. For example, previous work in PD patients has reported decreased activity of the SMA during gait [42] and diminished pre-movement electroencephalographic potentials over SMA prior to step initiation [43], [44]. Furthermore, Shine and colleagues [12] recently reported decreased fMRI neural activity within the STN and the MLR during freezing episodes, the magnitude of which was positively correlated with the severity of clinical freezing of gait. It is worth noting that a small number of fMRI [13] and PET imaging [45] studies have reported increased activity in the MLR in between FoG episodes providing evidence that neural activity during walking [42] or with imagined stepping [13] which may differ from neural activity during an actual freezing episode. That is to say, the increased activity in MLR during imagined normal gait [13] may be compensatory in-between FoG episodes when there is normal walking. With increased strain on the system (e.g. turning), this fragile and vulnerable compensation system (with decreased structural connectivity) fails, leading to FoG episodes [12].

Whereas studies of fMRI and PET provide insight into specific areas of activity during movement, resting state fcMRI allows for the assessment of integrated, spatially disparate neural networks. The current study allows for a greater understanding of how communication between these neural locomotor hubs is crucial for the appropriate coordination of posture with gait and the ability to adapt to changing environments that elicit freezing – e.g. turning, doorways, or crowded areas. We report that PD patients who experience freezing episodes have significantly greater communication between the SMA and the MLR and between the SMA and CLR than either FoG− or controls. The observed pattern of altered functional connectivity in FoG+ may be indicative of neural reorganization changes that may contribute to FoG rather than serving a compensatory role. We speculate that this altered pattern of connectivity within the locomotor circuitry may coincide with increased cognitive control of gait [3].

### Locomotor Network Structural Connectivity

The current results provide the first evidence of altered structural and functional connectivity of the locomotor network within the same cohort of PD patients. Structural connectivity between locomotor hubs was analyzed via probabilistic fiber tractography. Contrary to our initial hypothesis, both PD sub-groups had reduced structural connectivity along the hyperdirect rSTN – SMA loop, despite the significant group differences in functional connectivity between these same regions. It is also important to note that the structural connectivity loss is similar in both PD groups, even though the FoG− group had significantly shorter disease duration. While it appears that structural connectivity changes occur quickly between the STN – SMA in PD, the changes in functional connectivity do not appear to be linear. Similar to previous work [46] the current data show that early in PD progression (e.g. the FoG− group) there was an increase in functional communication from the STN to the SMA, whereas later in PD progression (the FoG+ group) there was a significant reduction in functional connectivity. Thus, although PD patients with and without FoG show similar structural decline along the hyperdirect loop between the rSTN – SMA, we speculate that FoG− patients compensate for this loss with significantly greater functional communication as we report here.

Consistent with right hemisphere involvement in response inhibition [47], we have recently demonstrated reduced structural connectivity of the pedunculopontine nucleus with the cerebellum, thalamus and multiple regions of the frontal cortex solely in the right hemisphere of patients with FoG+ [11]. Additionally, the more left hemisphere-lateralized the pedunculopontine nucleus tract volume in FoG+, the poorer the performance on cognitive tasks (Stroop task) requiring the initiation of appropriate actions and/or the inhibition of inappropriate actions [11]. Taken together there is mounting evidence speculating that deficits in conflict resolution (cognitive inhibition) is a principal component of freezing within PD [3].

### Cortical Contributions to FoG

Abnormal communication between cortical and subcortical structures in the presence of increased cognitive load has been proposed as a principal component underlying FoG [48]. Progression to more advanced PD (when FoG occurs) is likely associated with a progressive involvement of cortical structures such as the SMA [8]. Anticipatory postural adjustments that shift body weight to the stance leg for gait initiation are primarily mediated by the SMA [6], [49], [50]. It has been hypothesized that the SMA receives internal cues from the basal ganglia in order to coordinate and link together sub-movements (e.g. anticipatory postural adjustment and step initiation) [51]. As a result of basal ganglia dysfunction with PD progression, the SMA may lose its ability to sequence and coordinate postural adjustments and stepping. This is evidenced by studies demonstrating that anticipatory postural responses are smaller in PD [6] and may occur repeatedly without leading to a step [52]. In agreement with previous imaging work in humans [46] and electrophysiological recordings demonstrating excessive synchronization in basal ganglia-cortical circuitries in both humans [53], [54], [55] and animal PD models [56] we report greater functional connectivity in the rSTN – SMA loop in early stage PD patients (i.e. FoG−) than in controls. While previous work [46] suggests that overactivity of the hyperdirect pathway plays a crucial role in the pathophysiology of PD [55], [56], the current results suggest that the increased activity along this loop may serve a compensatory role in PD patients who do not experience FoG, whereas this compensation is lost over time, or was never present, in FoG+ individuals.

### Limitations

The current study has several limitations. Due to our strict motion correction procedures for processing of the functional imaging our sample size was noticeably reduced from the original cohort; however, when including all participants the results remain consistent (Figure S1). Further, for those participants included within the FoG− and FoG+ groups there is a noticeable difference in disease duration. This is often an issue when attempting to match disease characteristics between groups due to the onset of freezing episodes late in disease progression [57]. The increased disease duration provides the nervous system with more time to compensate for the disease-related changes, which may be reflected within our imaging results. Furthermore, early in disease progression, PD symptoms tend to be more asymmetric, possibly influencing the laterality differences seen in our FoG− group. This is tempered by the fact that both PD groups’ most affected side was evenly distributed and that the full cohort of data does not demonstrate laterality (Figure S1). It is also worth noting that the current study did not find significantly reduced structural connections between the rMLR – SMA in FoG+ patients as was found in recent work [11]. This is likely due to the smaller cohort investigated in the current study as a result of the strict motion correction processing implemented for the functional imaging.

## Conclusions

The current study provides the first evidence of altered structural and functional connectivity of the locomotor network within the same cohort of PD patients. These results demonstrate compromised structural integrity and abnormal functional communication between subcortical and cortical regions of the locomotor network in individuals who experience FoG. Increased functional connectivity between the SMA and the MLR and CLR may be reflective of a maladaptative compensation in FoG+. The loss of structural connectivity and the subsequent reduction in functional communication observed within FoG+ between the rSTN – SMA potentially represents a loss of the ability to inhibit competing motor programs (anticipatory postural adjustments) and to initiate the correct movement (gait). Because of the STN’s demonstrated association with inhibitory control the current findings add to the literature that implicates a role for executive dysfunction within the inhibitory domain leading to motor impairment in the form of FoG. The observed pattern of reduced structural and functional connectivity between the rSTN – SMA in a small sample of FoG+ patients, provides a specific target for cognitive neurorehabilitation [58] within these individuals.

## Supporting Information

Figure S1 Functional connectivity strength between the SMA and the other locomotor hubs for the full cohort of participants (HC = 15; FoG− = 12; FoG+ = 14). FoG+ patients show greater connectivity bilaterally to the MLR compared to HC and FoG−. FoG+ patients also demonstrated greater connectivity to the rCLR compared to FoG− and bilateral CLR compared to HC. FoG− patients had greater connectivity to the rSTN compared to HC and bilateral STN compared to FoG+. **P<0.001, *P<0.05.
(TIFF)
