# Supplementary figures and images for: Functional Reorganization of the Locomotor Network in Parkinson Patients with Freezing of Gait
Source: PLoS One. 2014 Jun 17;9(6):e100291. doi: 10.1371/journal.pone.0100291 (PMC4061081; doi:10.1371/journal.pone.0100291)

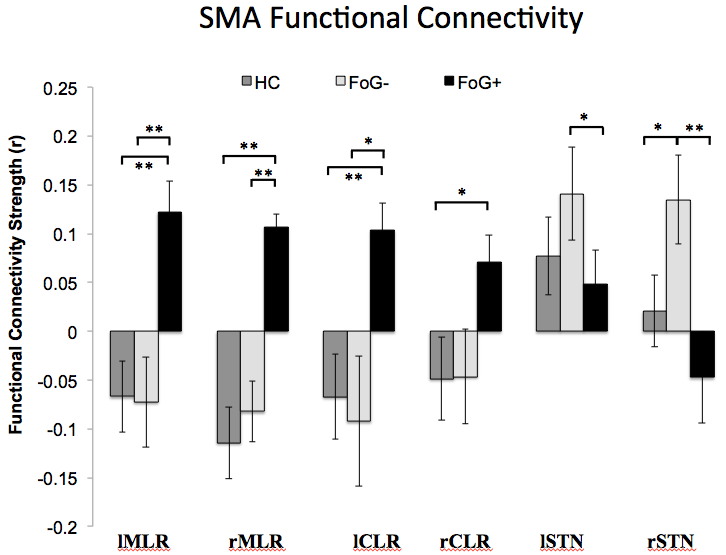

Supplement: Figure S1 — Functional connectivity strength between the SMA and the other locomotor hubs for the full cohort of participants (HC = 15; FoG− = 12; FoG+ = 14). FoG+ patients show greater connectivity bilaterally to the MLR compared to HC and FoG−. FoG+ patients also demonstrated greater connectivity to the rCLR compared to FoG− and bilateral CLR compared to HC. FoG− patients had greater connectivity to the rSTN compared to HC and bilateral STN compared to FoG+. **P<0.001, *P<0.05. (TIFF) [file pone.0100291.s001.tiff]
